# Differential Gene Expression Analysis Reveals Global LMTK2 Regulatory Network and Its Role in TGF-β1 Signaling

**DOI:** 10.3389/fonc.2021.596861

**Published:** 2021-03-18

**Authors:** Daniel F. Cruz, Nilay Mitash, Fangping Mu, Carlos M. Farinha, Agnieszka Swiatecka-Urban

**Affiliations:** ^1^Department of Nephrology, UPMC Children’s Hospital of Pittsburgh, University of Pittsburgh School of Medicine, Pittsburgh, PA, United States; ^2^Biosystems and Integrative Sciences Institute, Faculty of Sciences, University of Lisbon, Lisbon, Portugal; ^3^Center for Research Computing, University of Pittsburgh, Pittsburgh, PA, United States

**Keywords:** LMTK2, TGF-β1, RNAseq, gene expression, signaling pathway

## Abstract

Lemur tyrosine kinase 2 (LMTK2) is a transmembrane Ser/Thr kinase whose role has been increasingly recognized; however, when compared to other kinases, understanding of the LMTK2 networks and biological functions is still limited. Recent data have shown that transforming growth factor (TGF)-β1 plays a role in modulating LMTK2 function by controlling its endocytic trafficking in human bronchial epithelial cells. Here, we aimed to unveil the LMTK2 regulatory network and elucidate how it affects cellular functions and disease pathways in either TGF-β1 dependent or independent manner. To understand how the LMTK2 and TGF-β1 pathways interconnect, we knocked down (KD) LMTK2 using small(si)RNA-mediated silencing in human bronchial epithelial CFBE41o- cells, treated cells with TGF-β1 or vehicle control, and performed differential gene expression analysis by RNA sequencing (RNAseq). In vehicle-treated cells, LMTK2 KD affected expression of 2,506 genes while it affected 4,162 genes after TGF-β1 stimulation. Bioinformatics analysis shows that LMTK2 is involved in diverse cellular functions and disease pathways, such as cell death and survival, cellular development, and cancer susceptibility. In summary, our study increases current knowledge about the LMTK2 network and its intersection with the TGF-β1 signaling pathway. These findings will serve as basis for future exploration of the predicted LMTK2 interactions and signaling pathways.

## Introduction

Lemur tyrosine kinase 2 (LMTK2), also known as cyclin-dependent kinase-5 (CDK5)/p35 regulated kinase (cprk), kinase/phosphatase/inhibitor-2 (KPI2), brain-enriched kinase (BREK), and apoptosis-associated tyrosine kinase (AATYK)-2, is a member of the lemur family of membrane-anchored kinases. Despite its naming, LMTK2 is actually a Ser/Thr kinase that has been identified to interact with a restrict number of proteins, although related to different signaling pathways and key cellular processes [reviewed in (1)]. Earlier studies have shown that LMTK2 interacts with CDK5 and protein phosphatase 1 (PP1c) (2–5), cystic fibrosis transmembrane conductance regulator (CFTR) (6), myosin VI (7, 8), and anti- and pro-apoptotic proteins, such as B-cell lymphoma (BCL)2, BCL-xL and BCL2-interacting mediator of cell death (BIM) (9). These protein-protein interactions indicate that LMTK2 plays a role in diverse cellular functions, including protein transcription, intracellular protein trafficking, cell differentiation, and apoptosis (6–10).

LMTK2 is ubiquitously expressed in human tissues (11). Specifically, LMTK2 mRNA was detected in skeletal muscle, brain, and pancreas (5), while LMTK2 protein was detected in prostate and primary differentiated human bronchial epithelial (HBE) cells (6, 12). In polarized human bronchial epithelial cells, LMTK2 was detected at the apical and basolateral domains of the plasma membrane. At the apical membrane, LMTK2 co-immunoprecipitated with CFTR, phosphorylated its residue CFTR-Ser^737^, and facilitated CFTR endocytosis (6). The role of LMTK2 in endocytic trafficking was also supported by its interactions with myosin VI found to be necessary for transport of the transferrin receptor from early endosomes to the recycling vesicles (7, 8).

The crosstalk between the LMTK2 and transforming growth factor (TGF)-β1 signaling pathways was reported by two studies. First, LMTK2 was shown to phosphorylate kinesin-1 light chain-2 necessary for Smad2 signaling in HeLa cells (4). Smad2 is a transcription factor that mediates the canonical TGF-β1 signaling that regulates variety of cellular processes (13). Second, TGF-β1 recruited LMTK2 to the plasma membrane by a Rab11-dependent mechanism in human bronchial epithelial CFBE41o- cells (14), facilitating the LMTK2-mediated CFTR phosphorylation. These studies are clinically relevant because elevated TGF-β1 levels, observed in many CF patients, compromise the functional rescue of F508del-CFTR by current generation CFTR modulators (15).

The aim of this study was to elucidate a global LMTK2 regulatory network and its connection with the TGF-β1 signaling pathway in human bronchial epithelial cells. We identified candidate genes regulated by LMTK2 in both TGF-β1-dependent or independent manner. Based on the genes most affected by the LMTK2 knockdown (KD), we predict a complex LMTK2 regulatory network affecting a variety of cellular functions and disease pathways in TGF-β1-dependent and independent manner.

## Methods

### Cell Lines, Cell Culture and Reagents

Parental human bronchial epithelial CFBE41o- cells were cultured in Minimum Essential Medium (MEM; Thermo Fisher Scientific, Walthman, MA, USA), enriched with L-glutamine (Thermo Fisher Scientific), fetal bovine serum (FBS; Gemini Bio-Products, West Sacramento, CA, USA) and penicillin-streptomycin (Thermo Fisher Scientific) (6, 16, 17). CFBE41o- cells were seeded on collagen-coated 6-well plates (Corning, Corning, NY, USA), at a density of ~0.3x10^6^ cells. FBS was removed from the media 24h before experiments to avoid any residual TGF-β1, to increase cell polarization, and to promote cell cycle synchronization. Human TGF-β1 (Sigma-Aldrich) was resuspended in the vehicle containing 4 mM HCl and 1 mg/mL bovine serum albumin (BSA, Sigma-Aldrich) and used at a concentration 15ng/ml.

### RNA-Mediated LMTK2 KD

Transfection of CFBE41o- cells with siRNA targeting human LMTK2 gene (siLMTK2, siGENOME Human LMTK2 siRNA; Dharmacon, Cambridge, UK) or non-targeting siRNA (siCTRL, siGENOME NonTargeting Pool #2; Dharmacon) was performed using Lipofectamine RNAiMAX Transfection Reagent (Thermo Fisher Scientific), according to the manufacturer’s instructions. CFBE41o- cells were plated on collagen-coated cell culture plates and incubated with the optimized transfection mixture containing 50nM of siRNA, at 37°C for 24 h. Next day, culture medium was changed to remove FBS and transfection mixture. Experiments were conducted 2 days after siRNA transfection.

### Detection of LMTK2 Abundance by Western Blotting

Six biological replicates of CFBE41o- cells transfected with siCTRL or siLMTK2 were used to determine LMTK2 knockdown. After siRNA transfection, human bronchial epithelial CFBE41o- cells were cultured in 6-well plates for 2 days, and then lysed with a buffer containing 25mM HEPES, 10% v/v glycerol, 1% v/v Triton X-100, Complete Protease Inhibitor cocktail and PhosSTOP phosphatase inhibitor (Sigma-Aldrich). After centrifugation at 14,000xg for 10 minutes to pellet insoluble material, the soluble lysates were mixed with sample buffer (Bio-Rad Laboratories) with DL-dithiothreitol (DTT) and incubated at 37 ˚C for 30 minutes. Proteins were separated by SDS-PAGE using 7.5% pre-cast gels (Bio-Rad). LMTK2 was detected by western blotting (WB) with Western Lightning Chemiluminescence Reagent Plus (PerkinElmer LAS Inc., Boston, MA, USA), using previously validated polyclonal anti-LMTK2 antibody (#SAB4500900, Sigma-Aldrich, St. Louis, MO, USA) (14). Monoclonal anti-Ezrin (Cat. 610603, BD Biosciences, San Jose, CA, United States) was used as a loading control. Protein abundance was quantified by densitometry using exposures within the linear dynamic range of the film.

### Total RNA Sequencing

For gene expression analysis, we performed three biological replicates for each of the four conditions: (1) CFBE41o- cells transfected with siCTRL and treated with vehicle control; (2) CFBE41o- cells transfected with siCTRL and treated with TGF-β1; (3) CFBE41o- cells transfected with siLMTK2 and treated with vehicle control; and (4) CFBE41o- cells transfected with siLMTK2 and treated with TGF-β1. Total RNA was isolated using the Quick-RNA™ MiniPrep kit (Zymo Research, Irvine, CA, USA), according to the manufacturer’s instructions. The starting material, 1 μg of total RNA was used with the Illumina TruSeq Small RNA sample preparation kit (Illumina, San Diego, CA, USA). The RNA quantity and quality were assessed by Qubit 2.0 Fluorometer (Thermo Fisher Scientific) and Agilent Bioanalyzer Tapestation 2200 (Agilent Technologies, Santa Clara, CA, USA). In short, 3′ and 5’ adapters were ligated to total input RNA. Reverse transcription followed by polymerase chain reaction (PCR) was used to create cDNA constructs. This process selectively enriches those fragments that have adapter molecules on both ends. PCR was performed with primers that anneal to the ends of the adapters. cDNA libraries were then purified using QIAquick PCR purification kit (Qiagen). The cDNA libraries were validated using KAPA Biosystems primer premix kit with Illumina-compatible DNA primers and Qubit 2.0 fluorometer. Quality was examined using Agilent Bioanalyzer Tapestation 2200. The cDNA libraries were pooled at a final concentration of 1.8pM. Cluster generation and 75 bp paired-end read sequencing was performed on Illumina NextSeq 500’s (Illumina).

### RNA Sequencing Analysis

RNA sequencing reads were imported into CLC Genomics Workbench 11 (Qiagen, Valencia, CA, USA) and an initial quality control report of each read was performed. Adapter trimming from the sequence reads was performed using the following adapter sequences: AGATCGGAAGAGCACACGTCTGAACTCCAGTCA and AGATCGGAAGAGCGTCGTGTAGGGAAAGAGTGT.

Trimmed sequence reads mapping was performed using human GRCh38/hg38 as a reference genome and using the sequence and annotation from ENSEMBL version 86. Expression values were scaled to TPM and only mapped reads were considered. PCA plot was obtained and differential expression was performed using four different groups: (1) siCTRL-transfected cells with vehicle treatment, (2) siCTRL-transfected cells with TGF-β1 treatment, (3) siLMTK2-transfected cells with vehicle treatment, and (4) siLMTK2-transfected cells with TGF-β1 treatment. Pathway analysis was conducted using Ingenuity Pathway Analysis software (QIAGEN Bioinformatics), using two excluding criteria: max group mean (MGM) >1 and false discovery rate (FDR) p value <0.05. Two different bioinformatics analysis were performed: first, we compared the differential gene expression after LMTK2 knockdown in vehicle-treated cells (siCTRL-transfected cells with vehicle treatment versus siLMTK2-transfected cells with vehicle) treatment and then we evaluated gene expression after LMTK2 knockdown in TGF-β1-treated cells (siCTRL-transfected cells with TGF-β1 treatment versus siLMTK2-transfected cells with TGF-β1 treatment).

### Statistical Analysis

Within each condition, the gene lists were obtained using an independent Student’s t-test and fold-change analysis to identify upregulated and downregulated genes in siLMTK2-transfected CFBE41o- cells, relative to siCTRL. A significance threshold of P-value<0.05 was used to define modulated genes and signaling pathways of interest. A Venn diagram, obtained in http://bioinformatics.psb.ugent.be/webtools/Venn/, was used to illustrate genes being up- or downregulated simultaneously in both vehicle- and TGF-β1-treated CFBE41o- cells.

## Results

### LMTK2 Differentially Regulates Gene Expression After Stimulation With TGF-β1

**Figure 1:**
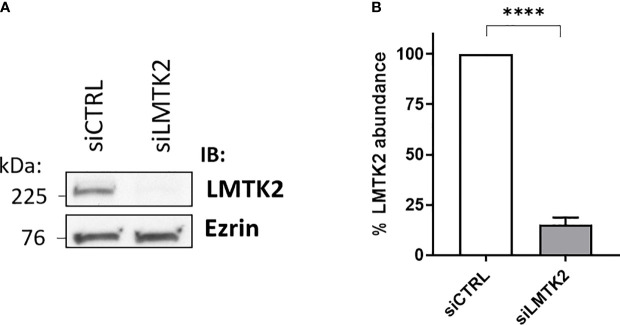
Western blot experiments demonstrating efficacy of the LMTK2 KD by siRNA. Representative blots **(A)** and summary of experiments **(B)** demonstrating that siLMTK2 decreased the LMTK2 protein abundance by 85%. CFBE41o- cells cultured in plastic dishes were transfected with siRNA against human LMTK2 (siLMTK2) or siRNA negative control (siCTRL). Ezrin was used as a loading control. ****p < 0.0001 versus siCTRL. 6 experiments/group. Error bars, S.E.M.

Our initial aim was to examine the LMTK2 KD using anti-LMTK2 siRNA. CFBE41o- cells were transfected with siLMTK2 or siCTRL and then LMTK2 protein abundance was assessed by WB. LMTK2 protein abundance was decreased by 85% in cells transfected with siLMTK2, compared to siCTRL (**Figure 1**). Previous studies have demonstrated that TGF-β1 stimulation does not affect total protein abundance of LMTK2 (14).

**Figure 2:**
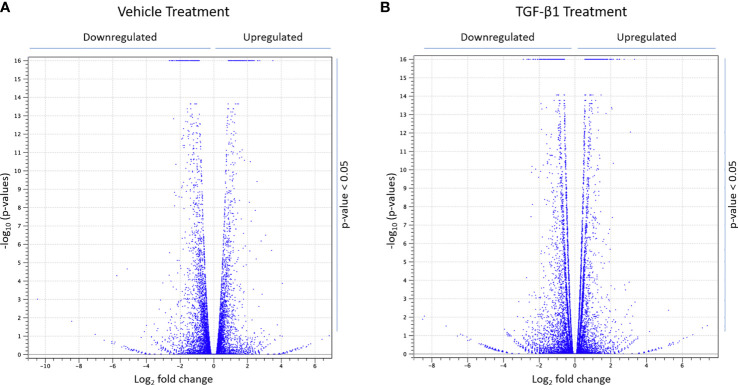
RNAseq data demonstrating the effects of LMTK2 KD on the mRNA landscape in the presence and absence of TGF-β1 stimulation. Volcano plot showing differential gene expression after LMTK2 KD in vehicle-treated **(A)** or TGF-β1-treated **(B)** CFBE41o- cells. CFBE41o- cells transfected with siLMTK2 or siCTRL were treated with vehicle control or TGF-β1 for 24h. Total RNA was isolated using Quick-RNA™ MiniPrep kit (Zymo Research) and quantified on Illumina NextSeq 500’s (Illumina). Data are expressed as Log2 fold change (FC) in cells transfected with siLMTK2, compared to siCTRL. N=3 for each condition.

Next, total RNA was isolated, from CFBE41o- cells transfected with siLMTK2 or siCTRL and treated with TGF-β1 or vehicle control for 24h, using the Quick-RNA™ MiniPrep kit (Zymo Research), and analyzed on the Illumina platform. The differential gene expression values were calculated from the RNAseq data, using CLC Genomics Workbench 11 software, and the expression levels of a total of 57,992 candidate genes, annotated in ENSEMBL version 86 human gene catalog, were assessed. In vehicle-treated CFBE41o- cells, LMTK2 KD changed the expression of 2,506 genes, upregulating 1,342 genes and downregulating 1,164 genes (**Figure 2A**). In TGF-β1-treated cells, LMTK2 KD affected the expression of 4,162 genes, of which 2,157 were upregulated and 2,005 were downregulated (**Figure 2B**). The above data demonstrate that under the vehicle control condition, LMTK2 affected the expression of 9.7% of the genes for which transcripts were identified (N = 2,506 out of 25,685) and after TGF-β1 stimulation, it affected 16.1% of the identified genes (N = 4,162 out of 25,832). Compared to vehicle control, TGF-β1 stimulation did not significantly change the proportion of genes that were up- or down-regulated by LMTK2 KD.

Next, we focused on the genes with the highest fold change after LMTK2 KD in CBFE41o- cells treated with vehicle or TGF-β1 (**Table 1**). Our RNAseq data confirm that LMTK2 expression was significantly decreased in cells transfected with siLMTK2, compared to siCTRL. *Cyclin-dependent kinase 6 (CDK6)* and *oxidized low-density lipoprotein receptor 1 (OLR1)* were significantly upregulated (> 6-fold change), while *torsin family 1 member B (TOR1B)*, *coiled-coil-helix-coiled-coil-helix domain containing 1 (CHCHD1)*, and *cellular repressor of E1A stimulated genes 1 (CREG1)* expression were significantly downregulated under both treatment conditions (> -5-fold change). *Fibroblast Growth Factor 7 Pseudogene 5 (FGF7P5)* was the most upregulated gene (> 160-fold change) in the TGF-β1-stimulated cells. In turn, AL358113.1, which encodes an uncharacterized protein, was the most downregulated gene (>-1,000-fold change) in vehicle-treated CFBE41o- cells. Many of the LMTK2 targets are involved in the regulation of important cellular mechanisms and distinct disease pathways, suggesting an important regulatory role of LMTK2.

**Table 1:** List of the genes most affected by LMTK2 KD in CFBE41o- cells treated with TGF-β1 or vehicle control.

Upregulation			Downregulation		
Gene	Fold-change	P-value	Gene	Fold-change	P-value
**Vehicle**					
CDK6	11.50	<10^-15^	AL358113.1	-1393.66	2.8x10^-5^
TTPAL	6.67	<10^-15^	TOR1B	-6.18	<10^-15^
OLR1	6.22	<10^-15^	LMTK2	-6.16	<10^-15^
ERVMER34-1	6.10	<10^-15^	CHCHD1	-5.84	<10^-15^
S1PR1	5.32	<10^-15^	CREG1	-5.54	<10^-15^
**TGF-β1**					
FGF7P5	169.31	2.2x10^-3^	LMTK2	-7.35	<10^-15^
CDK6	10.15	<10^-15^	CHCHD1	-6.34	<10^-15^
OLR1	6.82	<10^-15^	CREG1	-6.03	<10^-15^
GPAT3	6.78	<10^-15^	MMP9	-5.90	<10^-15^
TGM2	5.70	<10^-15^	TOR1B	-5.60	<10^-15^

CDK6, cyclin-dependent kinase 6; CHCHD1, coiled-coil-helix-coiled-coil-helix domain containing 1; CREG1, cellular repressor of E1A stimulated genes 1; ERVMER34-1, endogenous retrovirus group MER34 member 1; FC, fold change; FGF7P5, fibroblast growth factor 7 pseudogene 5; GPAT3, glycerol-3-phosphate acyltransferase 3; LMTK2, lemur tyrosine kinase 2; MMP9, matrix metallopeptidase 9; OLR1, oxidized low-density lipoprotein receptor 1; S1PR1, sphingosine-1-phosphate receptor 1; TGM2, transglutaminase 2; TOR1B, torsin family 1 member B; TTPAL, alpha tocopherol transfer protein like. Data are expressed as fold change (FC) in cells transfected with siLMTK2 versus siCTRL.

### LMTK2 Plays a Role in the Canonical TGF-β1 Signaling

**Figure 3:**
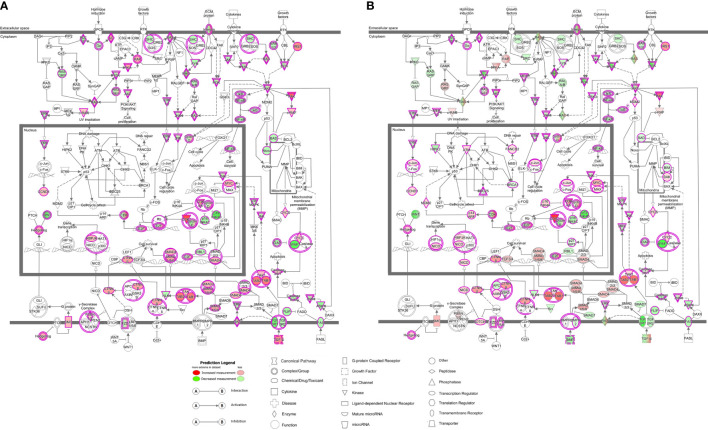
LMTK2 KD affects molecular mechanisms of cancer in vehicle- **(A)** or TGF-β1-treated **(B)** CFBE41o- cells. CFBE41o- cells transfected with siLMTK2 or siCTRL were treated with vehicle control or TGF-β1 for 24h. Total RNA was isolated using Quick-RNA™ MiniPrep kit (Zymo Research) and quantified on Illumina NextSeq 500’s (Illumina). LMTK2 knockdown changed the expression of 91 genes encoding mediators of molecular mechanisms of cancer signaling pathway in vehicle-treated CFBE41o- cells **(A)**, and this number increased to 142 genes after TGF-β1 treatment **(B)**. Image was obtained using the Ingenuity Pathway Analysis software.

**Figure 4:**
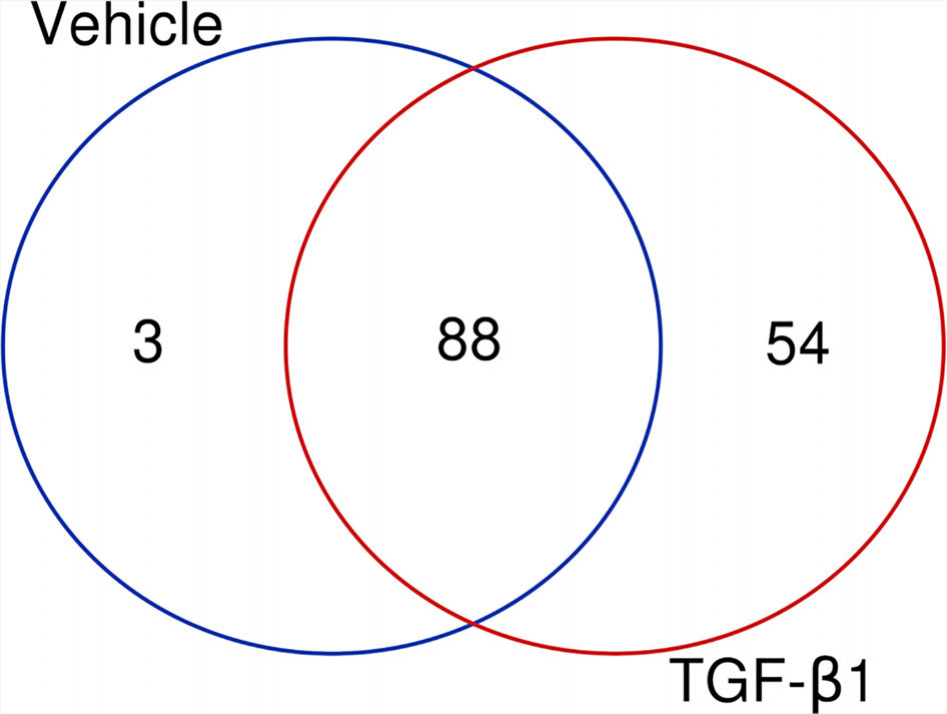
LMTK2 KD affects the expression of 88 genes in both vehicle- or TGF-β1-treated CFBE41o- cells. CFBE41o- cells transfected with siLMTK2 or siCTRL were treated with vehicle control or TGF-β1 for 24h. Total RNA was isolated using Quick-RNATM MiniPrep kit (Zymo Research) and quantified on Illumina NextSeq 500’s (Illumina). An overlap of 88 genes affected by LMTK2 KD was observed in cells treated with TGF-β1 or vehicle control. Vehicle treatment distinctly affected the expression of 3 genes, while TGF-β1 treatment compromised the regulation of 54 distinct genes. Venn diagram was obtained in http://bioinformatics.psb.ugent.be/webtools/Venn/.

Our next aim was to elucidate which signaling pathways were affected by LMTK2 KD and how TGF-β1 modulated the effects. The ‘molecular mechanisms of cancer’ pathway was highly affected by LMTK2 KD in both treatment groups (**Table 2**). Indeed, this observation corroborates several independent studies showing that LMTK2 is associated with the susceptibility and severity of colon, gastric, prostate and lung cancer (18–21). TGF-β1 affected higher number of genes in the ‘molecular mechanisms of cancer’ pathway, compared to vehicle control (142 and 91 genes for the TGF-β1 and vehicle treatment, respectively; **Table 2**, **Figure 3**). We observed an overlap of 88 genes affected by LMTK2 KD in cells treated with TGF-β1 or vehicle control (**Figure 4**). Of the 54 genes distinctly affected by TGF-β1 treatment after LMTK2 KD, the most downregulated were *Phosphoinositide-3-Kinase Regulatory Subunit 2 (PIK3R2)*, a lipid kinase that phosphorylates phosphatidylinositol, creating second messengers important in growth signaling, *Bone Morphogenetic Protein 8B (BMP8B)*, a secreted ligand of the TGF-β1 superfamily, and *Homeodomain Interacting Protein Kinase 2 (HIPK2)*, a Ser/Thr kinase involved in transcriptional regulation and apoptosis. These three genes are involved in the ‘molecular mechanisms of cancer’ pathway and are direct mediators of TGF-β1 signaling. These data demonstrate a previously unknown interplay between LMTK2 and TGF-β1 in the molecular mechanisms of cancer in airway epithelial cells.

**Table 2:** The signaling pathways most affected by LMTK2 KD in CFBE41o- cells treated with TGF-β1 or vehicle control.

Pathway	P-value	Gene (number)
**Vehicle**		
B Cell Receptor Signaling	4.68 x10^-14^	57/185 (30.8%)
Molecular Mechanisms of Cancer	3.42 x10^-13^	91/391 (23.3%)
HGF Signaling	2.28 x10^-11^	38/111 (34.2%)
PI3K Signaling in B Lymphocytes	3.90 x10^-11^	43/138 (31.2%)
NGF Signaling	5.65 x10^-11^	38/114 (33.3%)
**TGF-β1**		
Molecular Mechanisms of Cancer	5.10 x10^-19^	142/391 (36.3%)
Germ Cell-Sertoli Cell Junction Signaling	8.20 x10^-17^	77/171 (45.0%)
Senescence Pathway	3.43 x10^-15^	103/275 (37.5%)
EIF2 Signaling	4.36 x10^-15^	89/224 (39.7%)
IGF-1 Signaling	9.31 x10^-13^	50/104 (48.1%)

EIF2, eukaryotic initiation factor 2; HGF, hepatocyte growth factor; IGF-1, insulin-like growth factor 1; NGF, nerve growth factor; PI3K, phosphoinositide 3-kinase; TGF-β1, transforming growth factor β1. P-value (p <0.05) was obtained by comparing the expression levels of all genes that mediate these signaling pathways in CFBE41o- cells transfected with siLMTK2 versus siCTRL.

Other pathways affected by LMTK2 KD co-distributed with the treatment. In vehicle-treated CFBE41o- cells, LMTK2 KD affected the signaling pathways associated with B-cell receptor, hepatocyte growth factor (HGF), phosphoinositide 3-kinase (PI3K), and nerve growth factor (NGF). In TGF-β1-treated cells, LMTK2 KD affected the signaling pathways associated with germ cell-Sertoli cell junction, senescence, eukaryotic initiation factor 2 (EIF2), and insulin-like growth factor 1 (IGF-1). Taken together the above data indicate a specific role of LMTK2 in the global regulatory network and the modulatory effect of TGF-β1 on the LMTK2 function.

### LMTK2 and TGF-β1 Co-mediate Molecular Mechanisms and Cellular Functions

**Figure 5:**
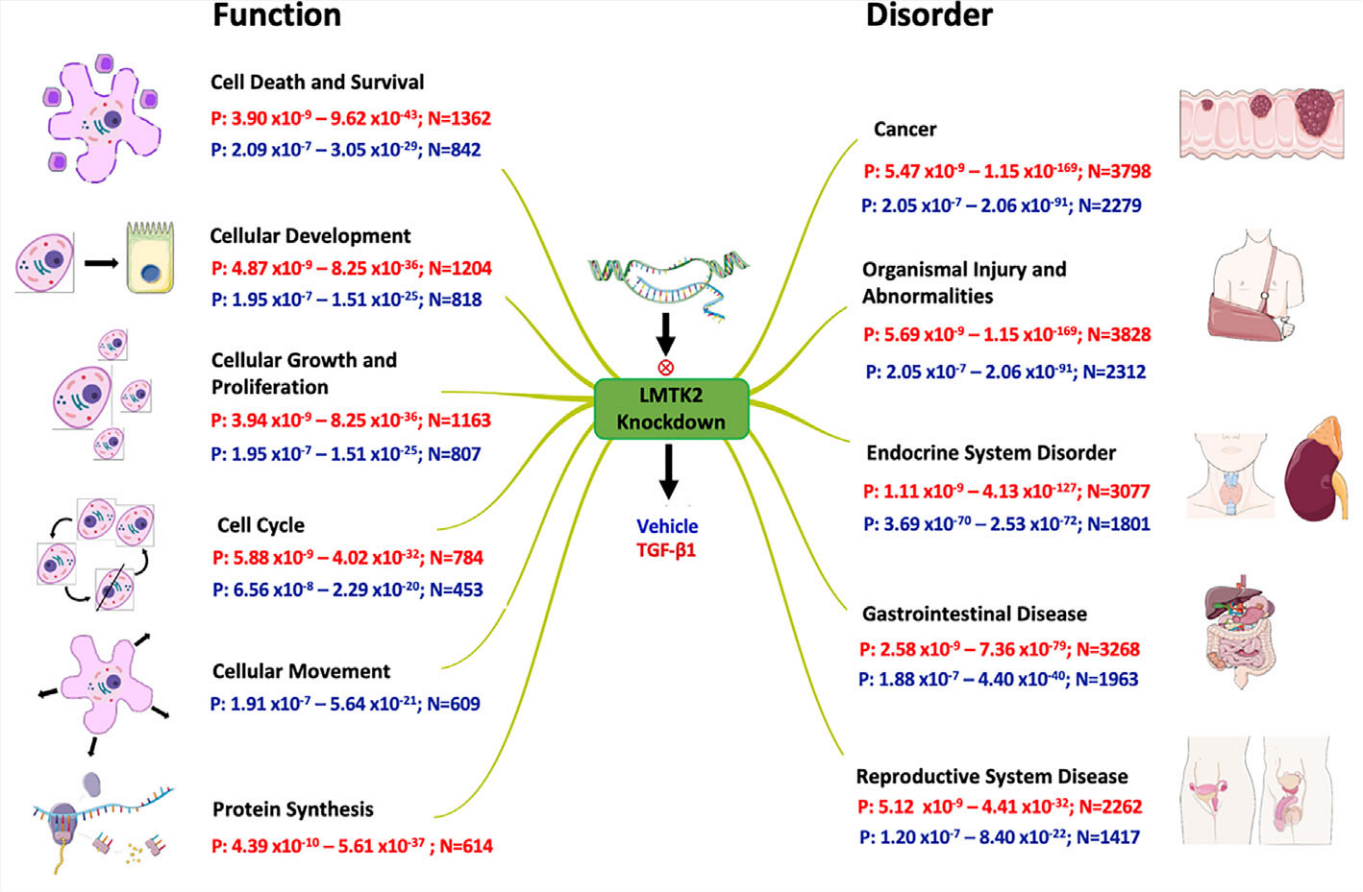
Effects of LMTK2 KD on cellular functions and organismal disorders. Shown are the ranges of p-value (P) and number of affected genes (N) in CFBE41o- cells treated with TGF-β1 (red) or vehicle control (blue). A different number of genes were affected by LMTK2 KD under the two experimental conditions. LMTK2 KD affected genes regulating protein synthesis only after TGF-β1 treatment. Genes controlling cell movement were affected by LMTK2 KD only in the absence of TGF-β1.

Next, we examined the molecular mechanisms and cellular functions associated with the differential gene expression after LMTK2 KD in CFBE41o- cells treated with TGF-β1 or vehicle control. We observed that LMTK2 KD affected the cell cycle, cell death and survival, cellular development, and cellular growth and proliferation (**Table 3**, **Figure 5**). TGF-β1 modulated regulation of these cellular functions by LMTK2. Indeed, the number of genes affected after LMTK2 KD increased in all mechanisms after TGF-β1 treatment. Furthermore, we observed that the cellular movement mechanisms were dependent on LMTK2 specifically in vehicle-treated cells, while the protein synthesis mechanisms were dependent on LMTK2 in TGF-β1-treated CFBE41o- cells.

**Table 3:** The cellular mechanisms most affected by LMTK2 KD in CFBE41o- cells treated with TGF-β1 or vehicle control.

Function	P-value range	Gene (number)
**Vehicle**		
Cell Death and Survival	2.09 x10^-7^ – 3.05 x10^-29^	842
Cellular Development	1.95 x10^-7^ – 1.51 x10^-25^	818
Cellular Growth and Proliferation	1.95 x10^-7^ – 1.51 x10^-25^	807
Cellular Movement	1.91 x10^-7^ – 5.64 x10^-21^	609
Cell Cycle	6.56 x10^-8^ – 2.29 x10^-20^	453
**TGF-β1**		
Cell Death and Survival	3.90 x10^-9^ – 9.62 x10^-43^	1362
Protein Synthesis	4.39 x10^-10^ – 5.61 x10^-37^	614
Cellular Development	4.87 x10^-9^ – 8.25 x10^-36^	1204
Cellular Growth and Proliferation	3.94 x10^-9^ – 8.25 x10^-36^	1163
Cell Cycle	5.88 x10^-9^ – 4.02 x10^-32^	784

P-value (p <0.05) was obtained by comparing the expression levels of all genes that mediate these cellular mechanisms in CFBE41o- cells transfected with siLMTK2 versus siCTRL.

### LMTK2 and TGF-β1 Co-mediate Diseases Pathways

Lastly, we aimed to examine which pathological processes and organ systems are more susceptible to LMTK2 KD and how it is modulated by TGF-β1 treatment. LMTK2 KD was associated with susceptibility to cancer, organismal injury, and endocrine, gastrointestinal and reproductive system diseases in the TGF-β1 and vehicle control group (**Table 4**, **Figure 5**). Although LMTK2 KD was associated with similar disorders in both treatment groups, TGF-β1 affected more genes associated with each disorder, suggesting that it changes the susceptibility to the disease pathways related to LMTK2.

**Table 4:** Pathological processes associated with LMTK2 KD in CFBE41o- cells treated with TGF-β1 or vehicle control.

Disorders	P-value range	Gene (number)
**Vehicle**		
Cancer	2.05 x10^-7^ – 2.06 x10^-91^	2279
Organismal Injury and Abnormalities	2.05 x10^-7^ – 2.06 x10^-91^	2312
Endocrine System Disorders	3.69 x10^-70^ – 2.53 x10^-72^	1801
Gastrointestinal Disease	1.88 x10^-7^ – 4.40 x10^-40^	1963
Reproductive System Disease	1.20 x10^-7^ – 8.40 x10^-22^	1417
**TGF-β1**		
Cancer	5.47 x10^-9^ – 1.15 x10^-169^	3798
Organismal Injury and Abnormalities	5.69 x10^-9^ – 1.15 x10^-169^	3828
Endocrine System Disorders	1.11 x10^-9^ – 4.13 x10^-127^	3077
Gastrointestinal Disease	2.58 x10^-9^ – 7.36 x10^-79^	3268
Reproductive System Disease	5.12 x10^-9^ – 4.41 x10^-32^	2262

P-value (p <0.05) was obtained by comparing the expression levels of all genes that mediate these pathological processes in CFBE41o- cells transfected with siLMTK2 versus siCTRL.

## Discussion

The RNAseq analysis in human bronchial epithelial cells elucidated effects of LMTK2 on gene expression and signaling pathways and demonstrated a complex interplay between the LMTK2 and TGF-β1 signaling pathways.

LMTK2 is an integral membrane protein associated with endosomal membranes (7, 14). Endocytic trafficking of membrane proteins can affect the intracellular signal transduction, including effects on the activity of various nuclear transcription factors. Indeed, LMTK2 has been previously shown to interact with different protein partners involved in several cell signaling events and protein transcription (3–5). LMTK2 activity was shown to promote the kinesin-1-mediated intracellular transport of Smad2, a transcription factor in the canonical TGF-β signaling pathway. LMTK2 interacts with Protein Phosphatase 1 (PP1c), leading to its phosphorylation and subsequent inactivation (3–5). In turn, PP1c inactivates the TGF-β1 signaling pathway, through inhibitory phosphorylation of type I TGF-β1 receptor. Our data provide novel evidence for the role of intracellular trafficking events regulated by LMTK2 in modulating the TGF-β1 signaling during cell death and survival, cellular development, and cancer susceptibility.

LMTK2 has been associated with several disorders, including CF (6), neurodegeneration (22–24), male infertility (25, 26) and different types of cancer (10, 18, 20, 21, 27). The role of LMTK2 in prostate cancer has been well established. LMTK2 was proposed as a potential biomarker and therapeutic target for prostate cancer (10). Indeed, *LMTK2* gene polymorphisms associated with decreased LMTK2 mRNA and protein levels were found in prostate cancer tissues (10, 19, 28). Furthermore, studies demonstrated that LMTK2 interacts with the androgen receptor (AR) and inhibits its activity (10, 12). The role of LMTK2 in other pathological processes, such as the lung, gastric, and colorectal cancer as well as infertility has not been well established. In this study, we demonstrated the role of LMTK2 in several signaling networks involved in the pathophysiology of these diseases.

Our results confirm the role of LMTK2 in cancer development and susceptibility. In fact, *LMTK2* polymorphisms that increase LMTK2 protein levels have been previously reported in cancer in different organs, including the lung (21, 29), gastrointestinal tract (18, 20), and prostate (19, 28, 30, 31). We observed that LMTK2 influenced the ‘molecular mechanism of cancer’ pathways and that treatment with TGF-β1 enhanced these effects. Indeed, the role of TGF-β1, a known mediator of cell growth and death, has also been investigated in many different cancer types. TGF-β1 acts primarily as a tumor suppressor, inhibiting cell proliferation and inducing apoptosis of premalignant epithelial cells (32, 33). However, at certain stages of carcinogenesis, TGF-β1 may function as a metastasis promoter, inducing epithelial-mesenchymal transition (EMT), cell invasion, and expression of genes that facilitate metastatic colonization of secondary sites (32, 33). Currently, it is unknown how and when TGF-β1 changes its function, from a suppressor to a promoter of cancer (32). For this reason, TGF-β1-directed therapies have several limitations (33). Besides ‘molecular mechanisms of cancer’, LMTK2 was also shown to regulate B cell receptor signaling, HGF signaling, PI3K signaling in B lymphocytes, IGF-1 signaling and senescence pathways, all of which have been previously described to play a role in cancer development (34–38). Thus, future studies may identify potential checkpoints of the LMTK2 pathway amenable for cancer therapeutic targeting.

Furthermore, we identified multiple molecular signaling pathways and cellular mechanisms that have been associated with distinct pathological disorders after LMTK2 KD. Signaling networks, such as NGF and eIF2, play a role in cellular development, growth, proliferation, and movement. Indeed, NGF signaling was previously shown to downregulate LMTK2 activity, leading to neurite outgrowth, which indicates that LMTK2 inhibits neuronal differentiation (39). Endogenous LMTK2 was found to be phosphorylated upon stimulation with NGF in PC12 cells through a protein kinase C (PKC)-dependent mechanism; however, a direct interaction between LMTK2 and PKC was not demonstrated (39). Our study increases the current knowledge on NGF-LMTK2 interaction, demonstrating that LMTK2 affects the NGF signaling homeostasis through direct regulation of 38 genes (33.3% of the signaling mediators), in vehicle-treated CFBE31o- cells.

Our data suggest that LMTK2 dysregulation may improve the susceptibility to several other pathologies, including organismal injury and abnormalities in the endocrine, gastrointestinal and reproductive systems. Our data shed light on the mechanisms of disease development. However, because our data were generated in human bronchial epithelial cells, further validation of the role of LMTK2 and TGF-β1 in the suggested disorders outside of human airway would be needed in the respective tissue models.

In summary, our data increase the knowledge on the interplay between LMTK2 and TGF-β1 regulatory networks in certain pathological processes affecting different organ systems, including distinct types of cancer. Thus, the study will help to direct future studies to validate the candidates as novel LMTK2 interactors, understand the role of LMTK2 in several disease processes, and identify novel treatments.

## Data Availability Statement

The RNAseq data have been deposited in NCBI’s Gene Expression Omnibus (GEO) and are accessible through GEO accession number GSE155987.

## Author Contributions

CF and AS-U designed the study, analyzed the data, and wrote and reviewed the manuscript. DC performed experiments, analyzed the data, and wrote and reviewed the manuscript. NM analyzed the data, and wrote and reviewed the manuscript. FM analyzed the data and reviewed the manuscript. All authors contributed to the article and approved the submitted version.

## Funding

This research was funded by the National Institutes of Health grants R01HL144539, R01HL090767, R01HL090767-02S1, and R56HL127202 (to AS-U), the Cystic Fibrosis Foundation SWIATE18G0 (to AS-U), the CFF RDP (to the University of Pittsburgh), and the UIDB/04046/2020 and UIDP/04046/2020 center grants from Fundação para a Ciência e a Tecnologia (FCT), Portugal (to BioISI). DC is a recipient of a fellowship from BioSys PhD programme (Ref. PD/BD/114384/2016) from FCT.

## Conflict of Interest

The authors declare that the research was conducted in the absence of any commercial or financial relationships that could be construed as a potential conflict of interest.

## Abbreviations

AATYK, apoptosis-associated tyrosine kinase; AR, androgen receptor; BCL, B-cell lymphoma; BIM, BCL2-interacting mediator of cell death; BMP8B, Bone Morphogenetic Protein 8B; CDK5, cyclin-dependent kinase 5; CDK6, cyclin-dependent kinase 6; CFTR, cystic fibrosis transmembrane conductance regulator; CHCHD1, coiled-coil-helix-coiled-coil-helix domain containing 1; CREG1, cellular repressor of E1A stimulated genes 1; EIF2, eukaryotic initiation factor 2; EMT, epithelial-mesenchymal transition; FDR, false discovery rate; FGF7P5, Fibroblast Growth Factor 7 Pseudogene 5; HGF, hepatocyte growth factor; HIPK2, Homeodomain Interacting Protein Kinase 2; IGF-1, insulin-like growth factor 1; LMTK2, Lemur tyrosine kinase 2; MGM, max group mean; NGF, nerve growth factor; OLR1, oxidized low-density lipoprotein receptor 1; PCR, polymerase chain reaction; PI3K, phosphoinositide 3-kinase; PIK3R2, Phosphoinositide-3-Kinase Regulatory Subunit 2; PKC, protein kinase C; PP1c, Protein Phosphatase 1; RNAseq, RNA sequencing; siCTRL, non-targeting siRNA; siLMTK2, siRNA targeting human LMTK2 gene; TGF-β1, transforming growth factor β1; TOR1B, torsin family 1 member B.
